# Multigene Typing of Croatian ‘*Candidatus* Phytoplasma Mali’ Strains

**DOI:** 10.3390/pathogens14100959

**Published:** 2025-09-23

**Authors:** Ivana Križanac, Martina Šeruga Musić, Jelena Plavec, Dijana Škorić

**Affiliations:** 1 Centre for Plant Protection, Croatian Agency for Agriculture and Food, Gorice 68B, HR-10000 Zagreb, Croatia; ivana.krizanac@hapih.hr (I.K.); jelena.plavec@hapih.hr (J.P.); 2 Department of Biology, Faculty of Science, University of Zagreb, Marulićev trg 9A, HR-10000 Zagreb, Croatia; martina.seruga.music@biol.pmf.unizg.hr

**Keywords:** apple proliferation, *Cacopsylla picta*, *aceF*, *pnp*, *imp*, *secY*, genotype, sequence type

## Abstract

Phytoplasmas (‘*Candidatus* Phytoplasma’) are intracellular pleomorphic plant pathogens belonging to the class Mollicutes. They colonize both plant hosts and insect vectors in their life cycle. Apple proliferation (AP) is one of the most important phytoplasmoses present in Europe, causing significant economic losses in apple production. The causal agent, ‘*Ca*. P. mali’, was identified in apple and *Cacopsylla picta* samples using both real-time PCR and nested PCR based on the amplification of 16S rDNA. The objective of this study was to gain deeper insights into the epidemiology of apple proliferation in Croatia. Variability of genetic markers other than *16S rRNA* was used for characterization of strains. Four molecular markers differing in level of conservation, *aceF*, *pnp*, *imp*, and *secY*, were selected in line with previously typed fruit tree phytoplasmas. New genotypes were discerned for each genetic marker, and 20 different sequence types were revealed in the Croatian strains of ‘*Ca*. P. mali’. On the basis of this comprehensive analysis, the founder sequence type ST1 (A13–P10–S12–I21) can be proposed. This is the first extensive research and multigene typing performed on Croatian ‘*Ca*. P. mali’ strains. Obtained results reveal considerable genetic diversity of epidemiological relevance limited to only two locations in north-western Croatia. Additionally, novel primers were constructed to amplify fragments larger than the entire coding region for all four genes in order to further expand the phytoplasma multi-locus sequence typing scheme.

## 1. Introduction

Phytoplasmas (‘*Candidatus* Phytoplasma’) are wall-less prokaryotes from the class Mollicutes that induce diseases in more than a thousand plant species worldwide [1]. They are limited to the phloem conducting elements in host plants, while in insect vectors they can be found in various organs and tissues, including the hemolymph and reproductive organs [1,2]. Their phylogeny is mainly based on the common bacterial phylogenetic marker *16S rRNA* gene [3]. Phytoplasma genomes are highly reduced and are among the smallest bacterial genomes described to date, regardless of their adaptation to both plant and insect hosts. Inability to cultivate phytoplasmas in axenic culture still hinders their detailed characterization as well as the acquisition of high-quality genomic DNA. Despite many difficulties, more than 50 draft or complete genomes have been sequenced in the last 20 years [4,5], with the first one being the OY-M strain of the subgroup 16SrI-B [6].

Fruit tree phytoplasmas occurring in Europe, ‘*Ca*. P. mali’, ‘*Ca*. P. pyri’ and ‘*Ca*. P. prunorum’, belong to the same phylogenetic cluster [7]. Their chromosomes are linear, which is an unusual feature for phytoplasmas and bacteria in general [8]. Multi-locus sequence typing (MLST) was proposed as a method applicable for characterization of many pathogenic bacteria [9]. This approach was used to differentiate phytoplasma strains and enhance understanding of the molecular epidemiology of European fruit tree phytoplasmas belonging to the ribosomal group 16SrX [10].

Characteristic symptoms of apple proliferation (AP) include the formation of “witches’ brooms”, enlarged stipules, and reduced fruit size with elongated pedicels [11]. The described symptoms were observed in apple orchards in north-western Croatia more than 40 years ago, and pleomorphic mycoplasma-like cells were detected in phloem tissues [12]. Although surveys of both ‘*Ca*. P. mali’ and ‘*Ca*. P. pyri’ presence in Croatian orchards started in 2003, ‘*Ca*. P. mali’, the causal agent of AP, was not confirmed in apple tree samples until 2011 [13,14]. Two psyllid species, *Cacopsylla picta* (Förster) [15] and *C. melanoneura* (Förster) [16], are identified as vectors of ‘*Ca*. P. mali’. Population dynamics of psyllids were monitored from 2005 to 2007, and both *C. melanoneura* and *C. picta* were found to be present and widespread [17]. With the availability of ‘*Ca.* P. mali’ genome [8], this study was designed to optimize the MLST scheme, enabling more accurate and robust molecular characterization of this phytoplasma. In parallel, it aimed to improve the understanding of apple proliferation epidemiology in Croatian apple orchards.

## 2. Materials and Methods

### 2.1. Plant and Insect Samples

Samples infected with ‘*Ca*. P. mali’ from a previous study, one *C. picta* and 60 apple tree samples, were used for MLST [14]. In 2016, an additional 176 *C. picta*, 34 *C. melanoneura*, and 12 apple tree samples were collected mainly in the western continental part of Croatia, where the AP disease pressure was the highest, with a particular focus on psyllid monitoring. Samples were tested for ‘*Ca*. P. mali’ using both real-time PCR [18] and nested PCR/RFLP methods [19,20,21] as previously described [14]. A total of 423 samples were tested for phytoplasma presence, of which 74 positive samples were used for MLST (Table S1).

### 2.2. MLST Primers and PCR Conditions

Primers designed to amplify the entire *aceF* gene were used for both direct PCR and sequencing, as previously described [14]. New primers were designed based on the available AP phytoplasma genome [8] to amplify fragments longer than complete coding regions of *imp*, *secY*, and *pnp* genes. Due to the amplicon size, additional primer pairs were needed for both nested PCR and sequencing for *pnp* and *secY* genes (Table 1). In a nested PCR reaction, 1 µL of direct PCR mix was used as a template. Both direct and nested PCR conditions for *aceF*, *pnp*, and *secY* amplification were the same: initial denaturation at 94 °C for 4 min, followed by 35 cycles of denaturation at 94 °C for 1 min, annealing at 52 °C for 2 min, extension at 68 °C for 3 min, and a final extension step of 7 min at 68 °C. To amplify the *imp* fragment in a direct PCR, the annealing and extension temperatures were 51 °C and 66 °C, respectively, with the same duration and number of cycles. Amplicons were custom sequenced (Macrogen Europe, Amsterdam, The Netherlands) on both strands for *aceF* and *imp*. For *secY*, additional internal primers were used for sequencing to increase coverage due to the amplicon size and to increase the coverage of the most variable region of the *secY* gene (Table 1).

### 2.3. Sequence Analysis

Raw nucleotide sequences were assembled and edited using both the Sequencher^®^ 4.7 software (Gene Codes Corporation, Ann Arbor, MI, USA, http://www.genecodes.com/) and Geneious 10.1.3 ([22], http://www.geneious.com), and aligned with ClustalX 2.0 [23]. A nucleotide BLAST search was performed on the NCBI website (https://blast.ncbi.nlm.nih.gov/Blast.cgi) against the GenBank core nucleotide database using the Megablast option optimized for highly similar sequences with default algorithm parameters. Consensus sequences of all four genes for each sample were concatenated using Geneious 10.1.3 [22] and analyzed using PhyloViz 2.0 [24]. Phylogenetic analyses were performed with MEGA 11 [25] using the maximum parsimony method with a bootstrap test (500 replicates) to support the inferred clades [26]. As amplicons and sequenced fragments exceeded the coding region for all four genes, both ExPasy [27] (http://web.expasy.org/translate/) and MEGA 11 [25] were used to identify open reading frames for each consensus sequence. Obtained sequences were trimmed in silico in two ways: first, to the length of reference sequences for each genotype of ‘*Ca*. P. mali’ [10] to enable comparison and continuation of the genotype labeling system, and second, to the length of the coding region to verify the accuracy of the consensus sequence. For each sample, a combination of *aceF*, *pnp*, *imp*, and *secY* genotypes was used to form a sequence type (ST), with the assigned ST number in the order of their prevalence.

## 3. Results

In a five-year period (2011–2014 and 2016), a total of 204 apple trees and 219 psyllid samples were analyzed using both nested PCR/RFLP and real-time PCR methods. Along with the previously published 61 positive samples [14], two apple and 11 *C. picta* samples were found to be infected with ‘*Ca*. P. mali’ in additional monitoring in 2016 (Table S1). None of the tested *C. melanoneura* harbored the phytoplasma. Out of 74 positive samples that were included in MLST, all four genetic markers were successfully typed for 64 positive samples (Table S2, Figure S1).

### 3.1. aceF, pnp, imp, and secY Genotyping

For the *aceF* fragment of approximately 790 bp in length, five of the eight previously described genotypes [10,28] were identified in Croatian samples, along with a new one labeled A27 (Table S2 and Figure S2). The predominant genotype, A13, was present in 67% of the analyzed samples. The unrooted phylogenetic tree obtained through the analysis of the full-length (1260 bp) *aceF* shows strong support for A27 genotype divergence (Figure S8).

Analysis of a 512 bp *pnp* gene fragment revealed seven distinct genotypes (Figure S3). Of the five previously described genotypes [10], only P9 and P10 are present in our samples, with P10 being the prevalent one in almost 70% of the samples (Table S2). Two new genotypes were identified, P17 and P18, with P18 found in only one apple sample (Figure S3 and Table S2). An unrooted phylogenetic tree based on the analysis of the complete *pnp* gene expectedly showed greater diversity because of the more than four times longer sequence (Figure S9).

Out of ten already described *imp* genotypes [10,28], five were found in Croatia together with six new ones (I36–I41). It is expectedly the most variable of the four typed genes (Figures S4 and S5, Table S2). The prevalent genotype, I21, was found in almost 50% of the samples (Table S2). A BLAST search of the newly identified genotype sequences against the GenBank nucleotide database revealed *imp* gene sequences identical to our new genotypes (Table S4). However, these entries had not been annotated and labeled according to the MLST scheme used in this study [29].

Analysis of nucleotide sequences of the *secY* gene fragment resulted in a phylogenetic tree separating into 12 genotypes (Figure S6). In addition to 7 known [10,28], 5 new genotypes (S17–S21) were identified in the samples from this study (Table S2). The genotype S12 was dominant and found in over 67% of the samples. Within the variable region of the *secY* gene, compared to the reference sequence of the strain AT ‘*Ca*. P. mali’ with a length of 1245 bp, there was either an insertion of three nucleotides (S17, sample 392) or deletions of multiple nucleotides (genotypes S10 and S12). As a result, the coding region length varies from 1227 bp (S11, S12, S19, and S18) to 1248 bp (S17). The *secY* gene fragment covers the most variable region (positions 397–438/1248 bp, Figure S7), and only a small number of significant nucleotide changes are found outside this section. Therefore, the unrooted phylogenetic tree based on the 1227–1248 bp *secY* gene sequence largely supports the tree obtained from the phylogenetic analysis of the shorter fragment (Figure S10).

Erroneously, labels I31 and S13 that had already been used for ‘*Ca*. P. pyri’ *imp* and *secY* genotypes from Azerbaijan [10] were used for the new genotypes in a more recent study [28]. Therefore, we propose correcting the genotype labels to avoid ambiguity, starting with the first available I35 and S16, respectively. These proposed labels are used throughout figures and tables in this study (Tables S2 and S4, Figures S4 and S6).

### 3.2. Sequence Types (STs) and Analysis of Concatenated Sequences

**Figure 1:**
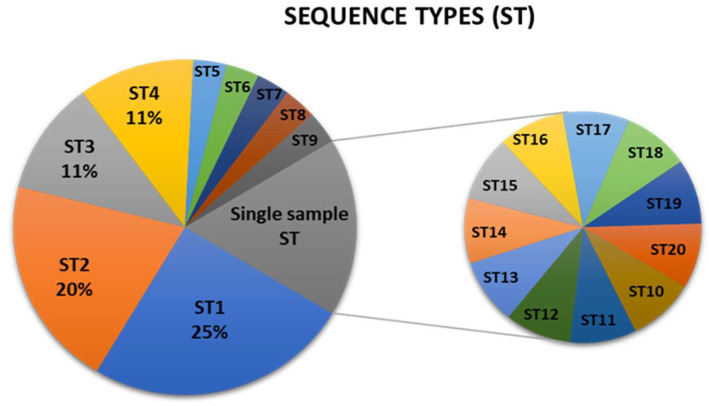
Prevalence of ‘*Ca*. P. mali’ sequence types (ST) in Croatian samples. MLST analysis based on the *aceF*, *pnp*, *imp*, and *secY* genotyping revealed 20 different sequence types. ST1 (A13–P10–I23–S12) is prevalent, and ST10–ST20 are represented in a single sample. Percentages are shown for 4 most frequent sequence types (ST1–ST4).

The obtained profile of genotype combinations for each sample was grouped to a sequence type (ST) and labeled in order of their frequency (Table S2). There are 20 different sequence types, with ST1 (A13–P10–I23–S12) represented in 25% of the Croatian samples (Figure 1). The ST4 (A15-P17-I21-S10) contains the new genotype P17 and is present in more than 10% of the samples. The ST2 profile (A13–P10–I21–S12) is the most common in *C. picta*, present in 50% of the insect samples (Figure 1, Table S2). Eleven out of 20 STs are represented in a single sample, mainly due to *imp* and *secY* variability, although ST11 and ST14 harbor known genotypes that are not frequent in Croatia (Figure 1, Table S2).

**Figure 2:**
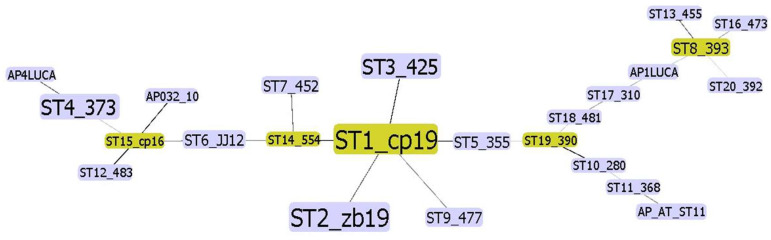
Graphical representation of concatenated sequences (ST) dataset analysis using Phyloviz 2.0. [24]. The size of a node represents the number of samples within the ST. Founder STs for each cluster are in green. Along with the sequence type label, a representative sample for the ST is shown for each node. Samples from a previous study with all four available sequences (AP_AT, AP1LUCA, AP032 10, and AP4 LUCA) are included in the analysis (Table S4).

**Figure 3:**
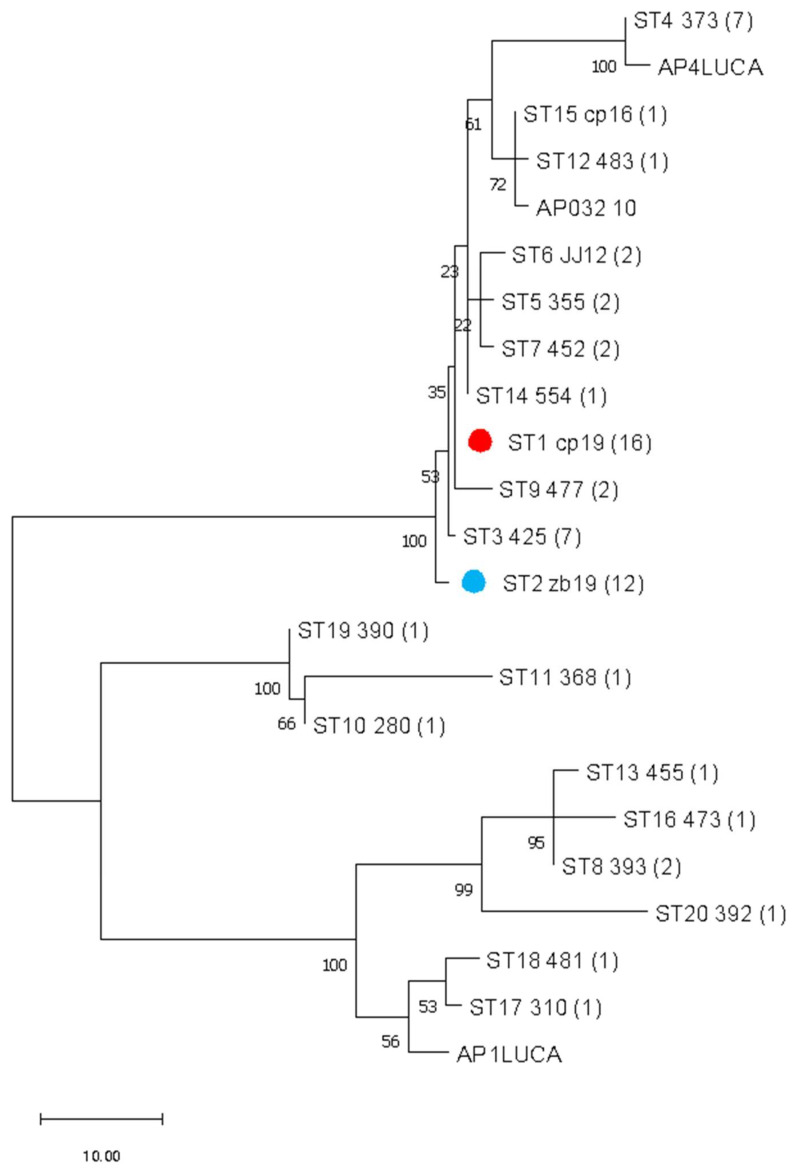
The unrooted phylogenetic tree was inferred using the maximum parsimony analysis of concatenated *aceF*, *pnp*, *imp*, and *secY* sequences (Table S2). A scale bar representing phylogenetic distance is given in the units of number of changes over the whole sequence. Numbers in brackets represent the number of samples for each sequence type (ST). Samples from a previous study with all four available sequences (AP1LUCA, AP032 10, and AP4 LUCA) are included in the analysis (Table S3). The percentage of replicate trees in which the associated taxa clustered together in the bootstrap test (500 replicates) is shown above the branches. The prevalent sequence type in overall samples, ST1, is marked with a red dot. The prevalent sequence type in *Cacopsylla picta* samples, ST2, is marked with a blue dot.

Concatenating all four gene sequences allowed simultaneous and comprehensive analysis of the ‘*Ca*. P. mali’ variability targeted in MLST. Analysis of concatenated sequences resulted in a finer resolution because all nucleotide differences are considered. In addition to interconnectedness shown in the phylogenetic network, the number of strains of the same profile is also considered and graphically represented by the node size (Figure 2). The frequency of each profile is used to select the founder ST for each cluster. Therefore, ST1 is considered an overall founder sequence type, and five clusters are suggested by analyzing this dataset (Figure 2). The unrooted phylogenetic tree inferred using the maximum parsimony analysis of concatenated *aceF*, *pnp*, *imp*, and *secY* sequences (Figure 3) largely supports the results represented in the network (Figure 2), with the presence of four main clusters.

## 4. Discussion

Following the results of a systematic survey of apple orchards, efforts were made to characterize Croatian *‘Ca*. *P.* mali’ strains in more detail in order to obtain a better insight into the epidemiology of this important plant pathogen. However, since the trees exhibiting symptoms reminiscent of apple proliferation were preferentially sampled, the percentage of infected apple trees in this study does not necessarily represent the overall apple infection rate in the country. The frequency of naturally infected psyllids, *C. picta* and *C. melanoneura*, as well as their ability to transmit the disease, differs significantly in countries and regions where the disease is present and well-studied [16,30]. In Croatia, *C. melanoneura* populations are more numerous than *C. picta*, and yet *C. picta* adults are present in apple orchards for a longer period before migration to overwintering coniferous hosts. This possibly increases their potential for phytoplasma acquisition [17]. Transovarial transmission of the phytoplasma to offspring has been proven for *C. picta* [31], thus bypassing the process of acquisition from the infected plant and multiplication in the vector. With more than 200 psyllid samples tested, we can positively conclude that the major AP phytoplasma vector in Croatia is *C. picta*. This vector infection pattern is consistent with research results from Germany, Switzerland, and France [32].

Primers developed in this study (Table 1) successfully amplified the target regions of all four MLST genes in the majority of samples. Novel genotypes were identified for each of the four genes analyzed. For *aceF*, genotype A13 was predominant (Figure S2 and Table S2), aligning with previous reports from strains collected in France, Italy, and Germany [10]. In ‘*Ca. P*. prunorum’, *aceF* genotypes A6 and A8 [10] have been associated with hypovirulent phytoplasma strains [33]. However, due to the lack of data on the biological properties of the Croatian ‘*Ca. P*. mali’ strains, it is currently not possible to infer similar associations from our findings. Phylogenetic analysis of the *pnp* gene fragment revealed two new genotypes—P17 and P18 (Figure S3 and Table S2). Genotype P17 was fairly frequent and is present in eight samples, while P18 was recorded in only one apple tree sample. The prevalent genotype P10 was present in almost 70% of the samples. From the results of genotyping European strains, the dominant genotype was P11 [10], which is not present in Croatia. When the entire coding region was analyzed, samples that were grouped within genotypes P9 and P10 separated into distinct branches (Figure S9), which is not surprising given that the complete coding region is four times longer than the fragment usually used in genotyping [10]. The *imp* gene was expectedly the most variable of the four typed genes (Figure S4, Table S2). This gene encodes the immunodominant membrane protein that is located on the cell surface and is thus exposed to positive selective pressure. Due to its role in interactions with both the apple and the insect vector, it is relevant in the molecular pathology of the disease [34,35]. Six novel genotypes (I36–I41) were identified in Croatia (Figure S4 and Table S2). This variability is common and consistent with studies of immunodominant membrane proteins from other phytoplasma species [35,36,37,38]. As many as 87 out of approximately 507 nucleotide positions in the *imp* gene sequence are variable, with the most significant difference being deletions within the sequence, causing the length to vary from 498 to 507 bp (Figure S5). The dominant genotype is I21, the same as at the European level [10,28]. The three genotypes identified in the largest number of samples (I21, I22, and I23) were grouped in the same cluster (Figure S4). Interestingly, all new genotypes were found exclusively in apple samples from two locations less than 5 km apart (Figure S1 and Table S2), while in *C. picta*, only I21 and I23 genotypes were present (Table S2). Changes in the *imp* gene in apple samples probably bring advantages, such as evading the host immune response [34]. In *C. picta*, phytoplasma must pass through various tissues and multiply to a sufficient concentration for successful transmission from salivary glands to the host [35,39,40]. This suggests that these two genotypes might be optimal for this translocation. The *secY* gene, which encodes the central subunit of the membrane transport system, has long been used as an additional phylogenetic marker for better differentiation of closely related phytoplasmas within the same 16S rRNA group [41]. Phylogenetic analyses of the obtained sequences show higher than expected variability (Figures S6 and S7, Table S2). Such variability of the *secY* gene is not straightforward and was not expected for this housekeeping gene, especially since MLST of the European fruit tree phytoplasma strains had shown that this gene is less variable than *aceF* and *pnp* [10]. The previously described genotype S12, which was dominant in ‘*Ca. P*. mali’ strains from five European countries [10], is present in by far the largest number of Croatian samples too (almost 70%) (Figure 1, Table S2). In this study, five new genotypes (S17–S21) were described (Figure S6 and Table S2). The same as with the *imp* gene, all new genotypes are present exclusively in apple samples from two locations in north-western part of Croatia (Figure S1 and Table S2). Introducing and naming new genotypes does not come without difficulties and we encountered inadvertently introduced erroneous labels for newly recorded genotypes for both *imp* and *secY* [28]. To ensure labeling accuracy moving forward, we propose reassigning the labels to the first available ones, namely I35 and S16, respectively.

Results of MLST revealed new genotypes and great diversity in two close-by locations in the north-western part of Croatia, Donji Mihaljevec and Sveta Marija (Figure S1 and Table S2). Apples have been traditionally grown in this region for a long time, and there are records indicating that ‘*Ca. P.* mali’ has been present here at least since the 1980s, when symptoms were first observed [12]. This long-lasting coevolution of the host and pathogen is one possible explanation for this diversification. Interestingly, this diversity is not represented in *C. picta* samples that harbor only one new genotype, P17 (Table S2). Results of genotyping individual genes were combined to form 20 different sequence types (Figure 1, Table S2). ST1 (A13–P10–I23–S12) is the most frequent one and is represented in 25% of the Croatian samples (Figure 1, Table S2). The profile ST2 (A13–P10–I21–S12) is the most common in *C. picta*, present in more than 50% of the insect samples (Figure 1, Table S2). Two sequence types differ only in two *imp* genotypes (I23 vs. I21). Since ST2 is dominant in *C. picta* samples, it can be hypothesized that I21 is significant in vector colonization and transmission. This is consistent with findings in Slovenia, where only the novel genotype I35 was found in the *C. melanoneura* sample [28]. Ten out of 20 STs are represented in a single sample, mainly due to *imp* and *secY* variability (Figure 1, Table S2).

Analysis of concatenated sequences suggested that the most frequent ST1 can be considered as the founder sequence type (Figure 1, Figure 2 and Figure 3). The unrooted phylogenetic tree inferred using the maximum parsimony analysis of concatenated *aceF*, *pnp*, *imp*, and *secY* sequences (Figure 3) largely supports the results represented in the network (Figure 2), with four main clusters. With only 18 previously fully genotyped strains from Austria, Switzerland, Germany, France, Italy, and Romania [10], 35 from neighboring Slovenia, and 64 as a part of this study, the dataset was still insufficient to create clear conclusions on the geographic distribution, origin, and virulence of individual strains. Nevertheless, the results of this study contribute to understanding the prevalence of individual genotypes in relation to the plant or insect host.

To improve and expand genotyping for phytoplasmas, it would be essential to agree on an MLST system for genes that have proven to be very informative, like *hflB* [42], SAP11 [43], or ribosomal protein (*rp*) genes [44]. The genome of ‘*Ca.* P. mali’ has been fully sequenced and annotated [8], enabling the search for additional, epidemiologically relevant, and evolutionary interesting genes. Furthermore, the creation of a database with representative genotype sequences according to uniform criteria and protocols would allow for the structured comparison of an increasing number of strains from across Europe. This would advance future applied research, such as the selection of apple cultivars resistant or tolerant to apple proliferation disease. The use of specific primers that allow amplification of the entire coding region for all four genes provided deeper insights into molecular characterization and may serve as a foundation for further in silico research of protein structure. Genotyping results are useful for understanding the epidemiology of phytoplasmas, and this study is a step toward creating a standardized MLST system for ‘*Ca*. P. mali’.

## 5. Conclusions

New genotypes are described in Croatian apple and *C. picta* samples for all four genes used in MLST: one for *aceF*, two for *pnp*, six for *imp*, and five for *secY* genes. All new *imp* and *secY* genotypes are found exclusively in apple samples. Twenty different sequence types (ST) are described, with 50% represented with only one sample, mainly due to *imp* and *secY* variability. Dominant ST1 was found in 25% of the samples, yet in *C. picta* samples, ST2 was present in more than half of the samples. Specific primers enabled amplification of the full coding region for all four genes, improving molecular characterization and supporting future in silico protein studies. The genotyping results provide valuable insights into the epidemiology of ‘*Ca.* P. mali’, with the dominance of ST1 highlighting its potential role in disease persistence and spread. This study contributes to the development of an expanded and improved MLST system for more precise characterization of phytoplasma strains.

## Figures and Tables

**Table 1:** Specific primers amplifying entire coding regions of ‘*Ca*. P. mali’ *aceF* *, *pnp*, *imp*, and *secY* (this study) genes used in MLST. Both amplicon size and coding region size are variable.

Gene	Method	Primer Name	Primer Sequence (5′–3′)	Amplicon Size (bp)	Coding Region (bp)
*aceF* *	PCR/ sequencing	acoB_F	CTGCTCCATCTAGAGTTAC	1600	1260
		lpd_R0m	GCTAGCTTTTATAGCAGCT		
*pnp*	PCR	pnpF	GCTCAGTTGGTAGAGCAT		
		pnpR	AGACACAAACACTACATACAT		
	Nested PCR/ sequencing	pnpF1	GGTAGAGCATCTGACTGTT	2400	2187
		pnpR1	CCCTCGATCGCCTTCTAT		
*imp*	PCR/ sequencing	impF	CGTAGAACCAAATGATAAAG	1000	507
		impR	GACATAGACATCGTTACGA		
*secY*	PCR	secYF1	CAGAGAATTCCTAAACGTG		
		secYR	GAATACCGTGAACAACTAC		
	Nested PCR/ sequencing	secYF2	GTTAATCTAGGTGCTTTAGA	1800	1227–1248
		secYR2	GTGAACAACTACTTCATTAAC		
	sequencing	secYF3	AGTTGGTGGCAATATTGA		
		secYR3	CTACTTCATTAACAGAAGTACA		

* Previously published primers [14].

## Data Availability

The consensus sequences presented in this study are openly available in NCBI GenBank at (https://www.ncbi.nlm.nih.gov/nucleotide/) with accession numbers listed in Table S3.

## Supplementary Materials

The following supporting information can be downloaded at https://www.mdpi.com/article/10.3390/pathogens14100959/s1, **Table S1.** Overall number of tested, positive and typed samples from previous (Križanac et al., 2017 [14]) and this study. **Table S2.** Genotyping results for all four loci (*aceF*, *pnp*, *imp* and *secY*), sequence types (ST) and their respective labels. Assigned new genotype labels from this study and sequence types represented with a single sample are in bold and shaded. Regions in Croatia are labeled CW (continental west), CE (continental east) and AD (Adriatic). **Table S3.** GenBank accession numbers of representative sequences from this study. Previously published sequences (Križanac et al., 2017 [14]) are shaded and new genotypes are in bold. **Table S4.** Sequences from GenBank used in this study. Reference sequences for ‘*Ca*. P. mali’ *aceF*, *pnp*, *imp* and *secY* genotypes are in bold and shaded (Danet et al. 2011 [10]). Sequences from GenBank blast search (https://blast.ncbi.nlm.nih.gov/Blast.cgi) identical to genotypes assigned in this study are underlined (Dermastia et al., 2018 [28]; Fránová et al., 2019 [45]; Kube et al., 2008 [8]; Seemüller et al., 2010 [29]). If sequence data is not available for a particular genetic marker, “X” indicates the absence of a corresponding sequence for that strain. **Figure S1.** Map of Croatia with sampling locations and dominant sequence type (ST) for each location. The sampling year and number of samples per location are listed in Table S2. **Figure S2.** Unrooted phylogenetic tree inferred using the maximum parsimony analysis of partial *aceF* gene sequences representative for each genotype (Tables S3 and S4). The percentage of replicate trees in which the associated taxa clustered together in the bootstrap test (500 replicates) are shown above the branches. Scale bar representing phylogenetic distance is given in the units of number of changes over the whole sequence. Genotypes present in Croatia are marked with an asterisk* and a new genotype, A27, is marked with a red triangle. **Figure S3.** Unrooted phylogenetic tree inferred using the maximum parsimony analysis of partial *pnp* gene sequences representative for each genotype (Tables S3 and S4). The percentage of replicate trees in which the associated taxa clustered together in the bootstrap test (500 replicates) are shown above the branches. Scale bar representing phylogenetic distance is given in the units of number of changes over the whole sequence. Genotypes present in Croatia are marked with an asterisk* and new genotypes, P17 and P18, are marked with red triangles. **Figure S4.** Unrooted phylogenetic tree inferred using the maximum parsimony analysis of *imp* gene sequences representative for each genotype (Tables S3 and S4). The percentage of replicate trees in which the associated taxa clustered together in the bootstrap test (500 replicates) are shown above the branches. Scale bar representing phylogenetic distance is given in the units of number of changes over the whole sequence. Genotypes present in Croatia are marked with an asterisk* and new genotypes, I36–I41, are marked with red triangles. **Figure S5.** Alignment of representative nucleotide sequences for each *imp* genotype (Tables S3 and S4) using ClustalX 2.1. The figure shows a highly variable region with deletions/insertions (positions 147–149; 203–205; 221–223/507), resulting in gene size ranging from 498 to 507 bp. **Figure S6.** Unrooted phylogenetic tree inferred using the maximum parsimony analysis of partial *secY* gene sequences representative for each genotype (Tables S3 and S4). The percentage of replicate trees in which the associated taxa clustered together in the bootstrap test (500 replicates) are shown above the branches. Scale bar representing phylogenetic distance is given in the units of number of changes over the whole sequence. Genotypes present in Croatia are marked with an asterisk* and new genotypes, S17–S21, are marked with red triangles. **Figure S7.** Alignment of representative nucleotide sequences for each *secY* genotype (Tables S3 and S4). The figure shows a highly variable region (397-438/1248) within which, in comparison to the reference sequence of the *secY* gene from the AT strain of *‘Ca*. P. mali’, either an insertion (genotype S17, sample 392) or a deletion (genotype S12, e.g., sample 489) occurred. **Figure S8.** Unrooted phylogenetic tree inferred using the maximum parsimony analysis of complete *aceF* gene sequences (Tables S2 and S3). The percentage of replicate trees in which the associated taxa clustered together in the bootstrap test (500 replicates) are shown above the branches. Scale bar representing phylogenetic distance is given in the units of number of changes over the whole sequence. Samples belonging to a novel A27 genotype are marked with red triangles. **Figure S9.** Unrooted phylogenetic tree inferred using the maximum parsimony analysis of complete *pnp* gene sequences (Tables S2 and S3). The percentage of replicate trees in which the associated taxa clustered together in the bootstrap test (500 replicates) are shown above the branches. Scale bar representing phylogenetic distance is given in the units of number of changes over the whole sequence. Samples belonging to novel P17 and P18 genotypes are marked with red triangles. **Figure S10.** Unrooted phylogenetic tree inferred using the maximum parsimony analysis of complete *secY* gene sequences (Tables S2 and S3). The percentage of replicate trees in which the associated taxa clustered together in the bootstrap test (500 replicates) are shown above the branches. Scale bar representing phylogenetic distance is given in the units of number of changes over the whole sequence. Samples belonging to novel S17–S21 genotypes are marked with red triangles.

## Abbreviations

The following abbreviations are used in this manuscript:

AP	Apple proliferation
MLST	Multi-locus sequence typing
ST	Sequence type
