# Supplementary material for: Multigene Typing of Croatian ‘Candidatus Phytoplasma Mali’ Strains
Source: Pathogens. 2025 Sep 23;14(10):959. doi: 10.3390/pathogens14100959 (PMC12567217; doi:10.3390/pathogens14100959)
Supplement: Supplementary file 1 [file pathogens-14-00959-s001.zip › pathogens-3847897-supplementary.pdf]

Supplementary materials

Table S1. Overall number of tested, positive and typed samples from previous (Križanac et al., 2017 [14]) and this study.

Sample (host)	No. tested	No. positive	No. typed
Malus domestica	204	62	52
Cacopsylla picta	180	12	12
Cacopsylla melanoneura	39	0	0
**total**	**423**	**74**	**64**

Table S2. Genotyping results for all four loci (*aceF*, *pnp*, *imp* and *secY*), sequence types (ST) and their respective labels. Assigned new genotype labels from this study and sequence types represented with a single sample are in bold and shaded. Regions in Croatia are labeled CW (continental west), CE (continental east) and AD (Adriatic).

Region	Sample label	Sampling year	Location	Host	*aceF*	*pnp*	*imp*	*secY*	Sequence type	ST label
CW	268	2014	Velika Ludina	Apple	A13	P10	I22	S12	A13-P10-I22-S12	ST3
	489	2011	Velika Ludina	Apple	A13	P10	I21	S12	A13-P10-I21-S12	ST1
	364	2012	Velika Ludina	Apple	A13	P10	I22	S12	A13-P10-I22-S12	ST3
	373	2013	Velika Ludina	Apple	A15	**P17**	I21	S10	A15-P17-I21-S10	ST4
	436	2013	Mičevec	Apple	A13	P10	I22	S12	A13-P10-I22-S12	ST3
	438	2013	Mičevec	Apple	A13	P10	I23	S12	A13-P10-I23-S12	ST2
	250	2014	Mičevec	Apple	A13	P10	I23	S12	A13-P10-I23-S12	ST2
	zb2	2016	Mičevec	*C. picta*	A15	**P17**	I21	S10	A15-P17-I21-S10	ST4
	zb3	2016	Mičevec	*C. picta*	A13	P9	I21	S12	A13-P9-I21-S12	ST5
	420	2011	Šušnjari	Apple	A13	P10	I23	S12	A13-P10-I23-S12	ST2
	441	2013	Šušnjari	Apple	A13	P10	I21	S12	A13-P10-I21-S12	ST1
	252	2014	Šušnjari	Apple	A13	P10	I23	S12	A13-P10-I23-S12	ST2
	JJ4	2016	Jastrebarsko	Apple	A15	**P17**	I21	S10	A15-P17-I21-S10	ST4
	JJ12	2016	Jastrebarsko	Apple	A15	P9	I21	S11	A15-P9-I21-S11	ST6
	Cp11	2012	Jastrebarsko	*C. picta*	A13	P10	I23	S12	A13-P10-I23- S12	ST2
	Cp13	2012	Jastrebarsko	*C. picta*	A15	**P17**	I21	S10	A15-P17-I21-S10	ST4
	Cp16	2012	Jastrebarsko	*C. picta*	A15	**P17**	I21	S11	A15-P17-I21-S11	**ST15**
	Cp17	2012	Jastrebarsko	*C. picta*	A15	P9	I21	S11	A15-P9-I21-S11	ST6
	Cp19	2012	Jastrebarsko	*C. picta*	A13	P10	I21	S12	A13-P10-I21-S12	ST1
	zb19	2016	Jastrebarsko	*C. picta*	A13	P10	I23	S12	A13-P10-I23-S12	ST2
	zb45	2016	Jastrebarsko	*C. picta*	A13	P10	I23	S12	A13-P10-I23-S12	ST2
	zb65	2016	Jastrebarsko	*C. picta*	A13	P10	I23	S12	A13-P10-I23-S12	ST2
	zb70	2016	Jastrebarsko	*C. picta*	A13	P10	I23	S12	A13-P10-I23-S12	ST2
	472	2013	Donji Mihaljevec	Apple	A13	P10	I23	S12	A13-P10-I23-S12	ST2
	473	2013	Donji Mihaljevec	Apple	A25	P10	**I39**	**S21**	A25-P10-I39- S21	**ST16**
	474	2013	Donji Mihaljevec	Apple	A13	P10	I21	S12	A13-P10-I21-S12	ST1
	475	2013	Donji Mihaljevec	Apple	A15	**P17**	I21	S10	A15-P17-I21-S10	ST4
	477	2013	Donji Mihaljevec	Apple	A26	P10	I32	S12	A26-P10-I32-S12	ST9
	276	2014	Donji Mihaljevec	Apple	A26	P10	I32	S12	A26-P10-I32-S12	ST9
	481	2011	Sveta Marija	Apple	**A27**	P9	**I36**	**S19**	A27-P9-I36- S19	**ST18**
	483	2011	Sveta Marija	Apple	A13	**P17**	I21	S11	A13-P17-I21-S11	**ST12**
	519	2011	Sveta Marija	Apple	A15	**P17**	I21	S10	A15-P17-I21-S10	ST4
	310	2012	Sveta Marija	Apple	**A27**	P10	**I41**	**S19**	A27-P10-I41- S19	**ST17**
	389	2012	Sveta Marija	Apple	A16	P10	**I39**	**S20**	A16-P10-I39-S20	ST8
	390	2012	Sveta Marija	Apple	A13	P9	**I37**	**S18**	A13-P9-I37- S18	**ST19**
	392	2012	Sveta Marija	Apple	A16	P10	**I38**	**S17**	A16-P10-I38- S17	**ST20**
	393	2012	Sveta Marija	Apple	A16	P10	**I39**	**S20**	A16-P10-I39-S20	ST8
	448	2013	Sveta Marija	Apple	A13	P10	I21	S12	A13-P10-I21-S12	ST1

Region	Sample label	Sampling year	Location	Host	*aceF*	*pnp*	*imp*	*secY*	Sequence type	ST label
CW	449	2013	Sveta Marija	Apple	A13	P10	I21	S12	A13-P10-I21-S12	ST1
	450	2013	Sveta Marija	Apple	A13	P10	I21	S12	A13-P10-I21-S12	ST1
	452	2013	Sveta Marija	Apple	A15	P9	**I40**	S12	A15-P9-I40-S12	ST7
	453	2013	Sveta Marija	Apple	A15	P9	**I40**	S12	A15-P9-I40-S12	ST7
	455	2013	Sveta Marija	Apple	A13	**P18**	**I39**	**S20**	A13-P18-I39-S20	**ST13**
	456	2013	Sveta Marija	Apple	A13	P10	I21	S12	A13-P10-I21-S12	ST1
	457	2013	Sveta Marija	Apple	A13	P10	I21	S12	A13-P10-I21-S12	ST1
	458	2013	Sveta Marija	Apple	A13	P10	I21	S12	A13-P10-I21-S12	ST1
	459	2013	Sveta Marija	Apple	A13	P10	I21	S12	A13-P10-I21-S12	ST1
	460	2013	Sveta Marija	Apple	A13	P10	I21	S12	A13-P10-I21-S12	ST1
	461	2013	Sveta Marija	Apple	A13	P10	I21	S12	A13-P10-I21-S12	ST1
	6K	2013	Sveta Marija	*C. picta*	A13	P10	I23	S12	A13-P10-I23-S12	ST2
	277	2014	Sveta Marija	Apple	A13	P10	I22	S12	A13-P10-I22-S12	ST3
	278	2014	Sveta Marija	Apple	A13	P10	I21	S12	A13-P10-I21-S12	ST1
	279	2014	Sveta Marija	Apple	A13	P10	I21	S12	A13-P10-I21-S12	ST1
	280	2014	Sveta Marija	Apple	A13	P9	I24	**S18**	A13-P9-I24- S18	**ST10**
CE	354	2011	Osijek	Apple	A13	P10	I23	S12	A13-P10-I23-S12	ST2
	355	2011	Osijek	Apple	A13	P9	I21	S12	A13-P9-I21-S12	ST5
	356	2011	Osijek	Apple	A13	P10	I22	S12	A13-P10-I22-S12	ST3
	357	2011	Osijek	Apple	A13	P10	I23	S12	A13-P10-I23-S12	ST2
	391	2013	Osijek	Apple	A15	**P17**	I21	S10	A15-P17-I21-S10	ST4
	411	2013	Osijek	Apple	A13	P10	I21	S12	A13-P10-I21-S12	ST1
	368	2012	Staro Petrovo selo	Apple	A13	P9	I24	S9	A13-P9-I24-S9	**ST11**
	554	2011	Žubrica	Apple	A15	P10	I21	S12	A15-P10-I21-S12	**ST14**
	425	2013	Žubrica	Apple	A13	P10	I22	S12	A13-P10-I22-S12	ST3
AD	493	2011	Opuzen	Apple	A13	P10	I22	S12	A13-P10-I22-S12	ST3

Table S3. GenBank accession numbers of representative sequences from this study. Previously published sequences (Križanac et al., 2017 [14]) are shaded and new genotypes are in bold.

Gene	Genotype label	Sample label	Acc. No.
aceF (complete cds)	A13	493	KU644715
		354	KU644726
		355	KU644725
		356	KU644724
		357	KU644723
		489	KU644729
		364	KU644722
		420	KU644717
		483	KU644728
		6K	KT325594
		390	KU644720
		368	KU644721
	A15	554	KU644714
	A16	392	KU644719
		393	KU644718
	A25	473	MF489187
	A26	477	MF489185
		276	MF489186
	**A27**	310	KU644716
		481	KU644727
pnp	**P17**	391	MF489193
		373	MF489188

Gene	Genotype label	Sample label	Acc. No.
(512 bp fragment)	**P17**	zb2	MF489189
		JJ4	MF489190
		cp13	MF489191
		cp16	MF489192
		475	MF489194
		483	MF489195
		519	MF489196
	**P18**	455	MF489197
pnp (complete cds)	P10	356	PX278437
		268	PX278438
		425	PX278439
		310	PX278440
		6K	PX278441
	P9	368	PX278442
		390	PX278443
		452	PX278444
		Cp17	PX278445
		481	PX278446
		355	PX278447
		zb3	PX278448
	**P17**	391	PX278449
		Cp16	PX278450
	**P18**	455	PX278451
imp	I32	477	MF489199
		276	MF489200
	**I36**	481	MF489201
	**I37**	390	MF489204
	**I38**	392	MF489205
	**I39**	455	MF489209
		473	MF489198
		393	MF489206
		389	MF489203
	**I40**	452	MF489207
		453	MF489208
	**I41**	310	MF489202
secY (646-667 bp fragment)	**S19**	481	MF489211
		310	MF489212
	**S20**	389	MF489213
		393	MF489216
		455	MF489217
	**S21**	473	MF489210
	**S17**	392	MF489215
	**S18**	390	MF489214
		280	MF489218
secY (complete cds)	S9	368	PX278429
	S10	391	PX278430
	S11	483	PX278431
	S12	493	PX278432
		252	PX278433
		449	PX278434
		489	PX278435
		411	PX278436
	**S17**	392	PX278427
	**S18**	390	PX278428

Gene	Genotype label	Sample label	Acc. No.
	**S19**	481	PX278424
	**S20**	393	PX278425
	**S21**	473	PX278426

Table S4. Sequences from GenBank used in this study. Reference sequences for '*Ca*. P. mali' *aceF*, *pnp*, *imp* and *secY* genotypes are in bold and shaded (Danet et al. 2011 [10]). Sequences from GenBank blast search (https://blast.ncbi.nlm.nih.gov/Blast.cgi) identical to genotypes assigned in this study are underlined (Dermastia et al., 2018 [28]; Fránová et al., 2019 [45]; Kube et al., 2008 [8]; Seemüller et al., 2010 [29]). If sequence data is not available for a particular genetic marker, "X" indicates the absence of a corresponding sequence for that strain.

(Reference) strain	*aceF*	Acc. no.	*pnp*	Acc. no.	*imp*	Acc. no.	*secY*	Acc. no.
AP-AT	**A13**	FN598184	**P9**	FN598200	**I24**	FN600733	**S9**	FN598213
AP13	**A14**	FN598185	P11	X	**I22**	FN600731	S12	X
AP032-10	**A15**	FN598186	**P12**	FN598203	**I21**	FN600730	**S11**	FN598215
AP1Luca	**A16**	FN598187	**P13**	FN598204	**I25**	FN600734	S12	X
TN/1	**A22**	FN598188	X	X	X	X	X	X
NW/2	**A23**	FN598189	X	X	I24	X	X	X
AP15	A13	X	**P10**	FN598201	**I23**	FN600732	**S12**	FN598216
AP28	A13	X	**P11**	FN598202	X	X	S9	X
AP4Luca	A15	X	P12	X	I22	X	**S10**	FN598214
TN2	X	X	X	X	**I29**	FN600735	X	X
HD/1	X	X	X	X	**I30**	FN600736	X	X
32_2014	X	X	X	X	X	X	S20	KT906170
3/93-2.6b	X	X	X	X	I32	FN658475	X	X
3/93-2.6a	X	X	X	X	I37	FN658476	X	X
D401/12	A25	MG972416	X	X	X	X	S15	MG972439
D201/12	A26	MG972416	X	X	I32	MG972427	X	X
D48-2/15	X	X	X	X	I35 (I31*)	MG972426	X	X
D940/13	X	X	X	X	I33	MG972428	S16 (S13*)	MG972437
D142/14	X	X	X	X	X	X	S14	MG972438
AP-AT complete chromosome		CU469464		AP-AT complete sequence		NC_011047		

*Genotypes (Dermastia et al. 2018 [28]) with the proposed changes in the label.

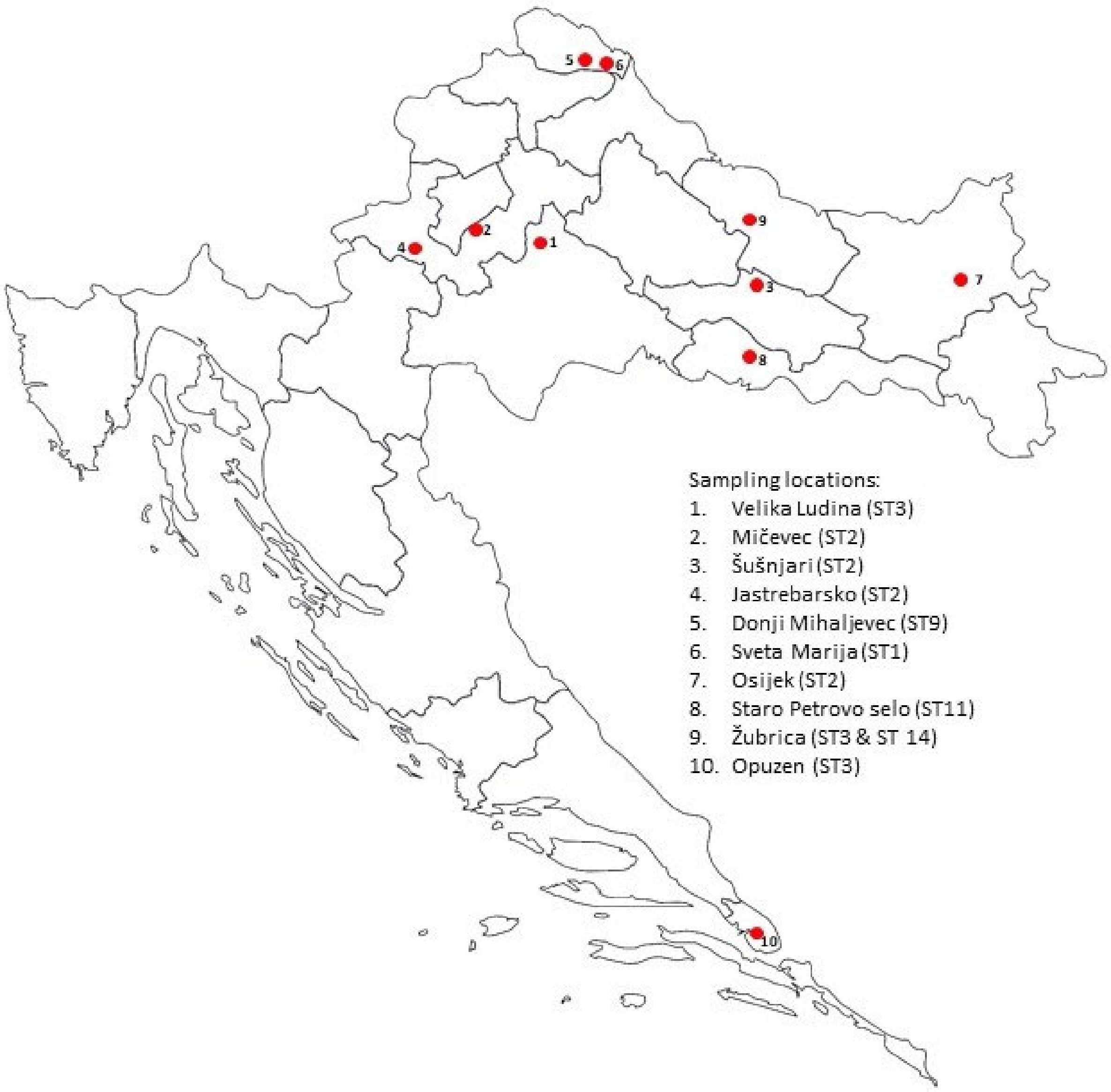

Figure S1. Map of Croatia with sampling locations and dominant sequence type (ST) for each location. The sampling year and number of samples per location are listed in Table S2.

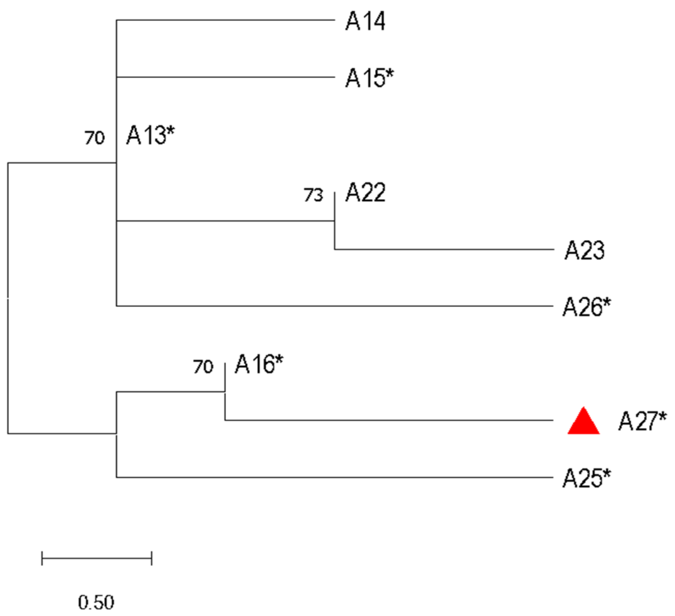

Figure S2. Unrooted phylogenetic tree inferred using the maximum parsimony analysis of partial *aceF* gene sequences representative for each genotype (Tables S3. and S4.). The percentage of replicate trees in which the associated taxa clustered together in the bootstrap test (500 replicates) are shown above the branches. Scale bar representing phylogenetic distance is given in the units of number of changes over the whole sequence. Genotypes present in Croatia are marked with an asterisk* and a new genotype, A27, is marked with a red triangle.

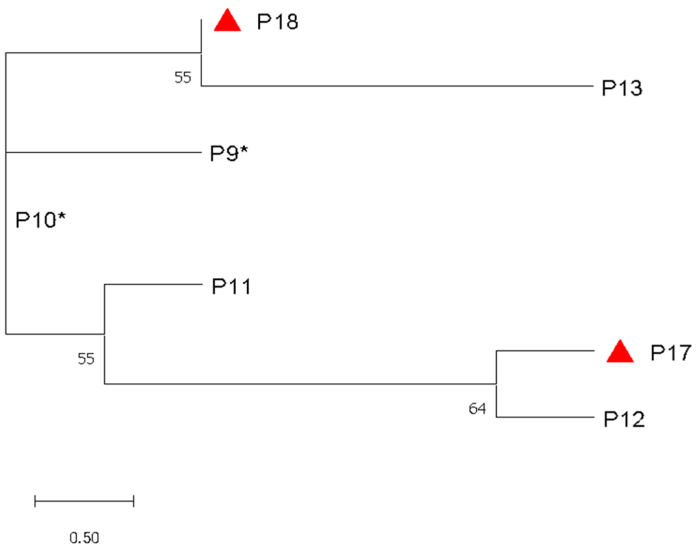

Figure S3. Unrooted phylogenetic tree inferred using the maximum parsimony analysis of partial *pnp* gene sequences representative for each genotype (Tables S3. and S4.). The percentage of replicate trees in which the associated taxa clustered together in the bootstrap test (500 replicates) are shown above the branches. Scale bar representing phylogenetic distance is given in the units of number of changes over the whole sequence. Genotypes present in Croatia are marked with an asterisk* and new genotypes, P17 and P18, are marked with red triangles.

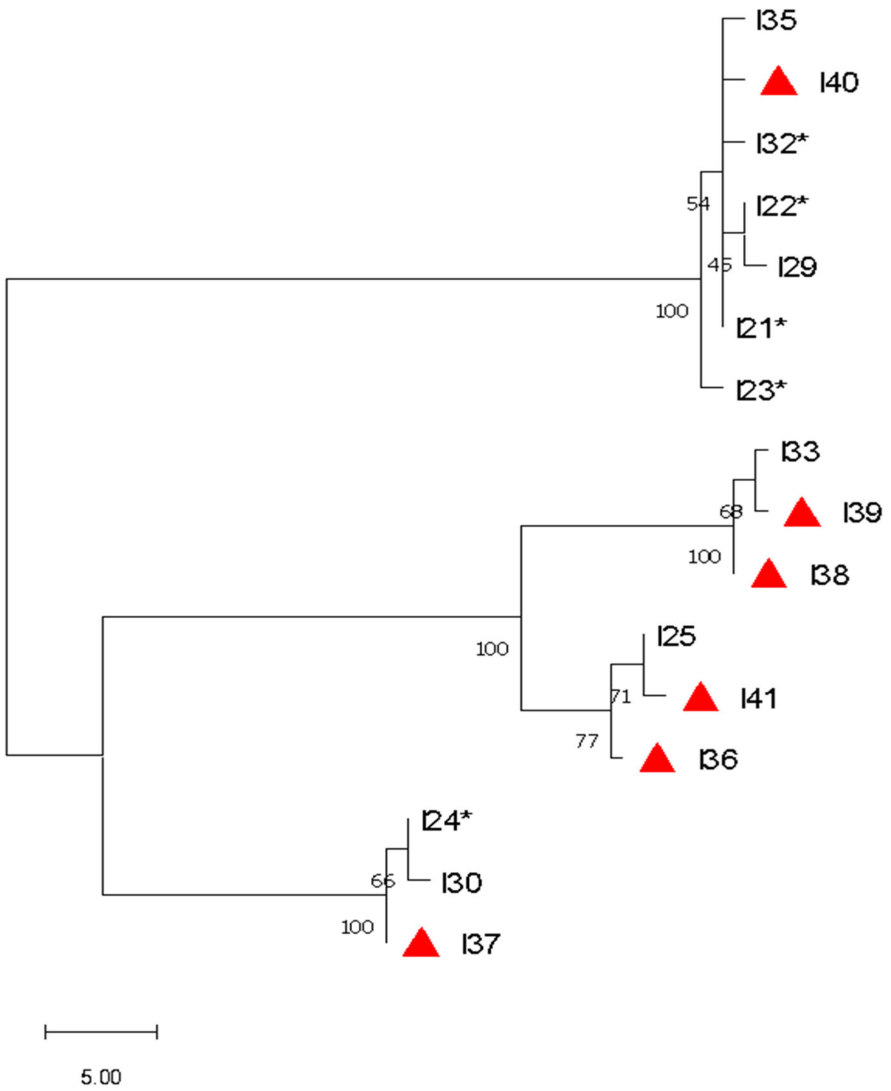

Figure S4. Unrooted phylogenetic tree inferred using the maximum parsimony analysis of *imp* gene sequences representative for each genotype (Tables S3. and S4.). The percentage of replicate trees in which the associated taxa clustered together in the bootstrap test (500 replicates) are shown above the branches. Scale bar representing phylogenetic distance is given in the units of number of changes over the whole sequence. Genotypes present in Croatia are marked with an asterisk* and new genotypes, I36 – I41, are marked with red triangles.

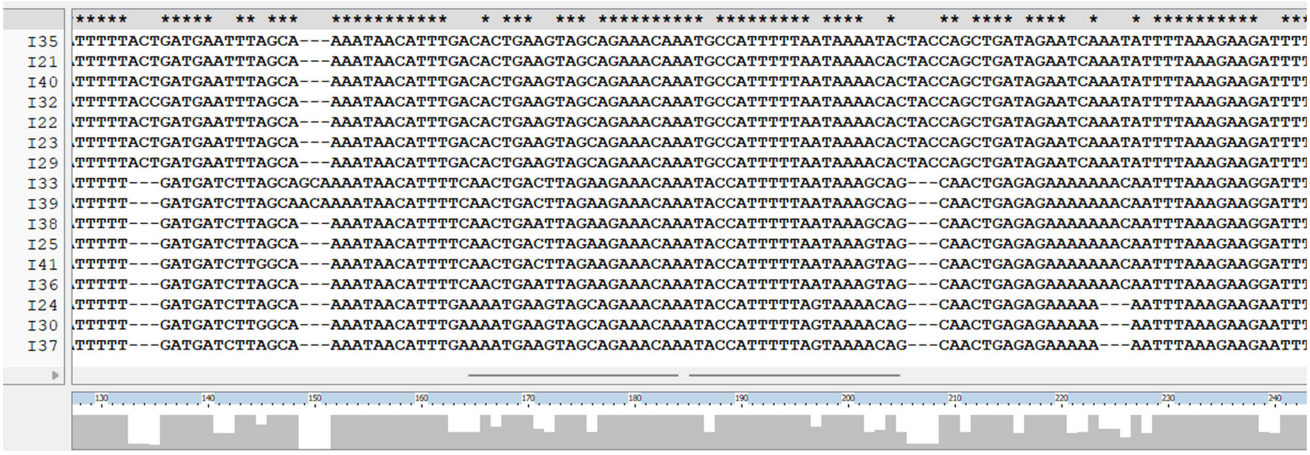

Figure S5. Alignment of representative nucleotide sequences for each *imp* genotype (Tables S3. and S4.) using ClustalX 2.1. The figure shows a highly variable region with deletions/insertions (positions 147–149; 203–205; 221–223/507), resulting in gene size ranging from 498 to 507 bp.

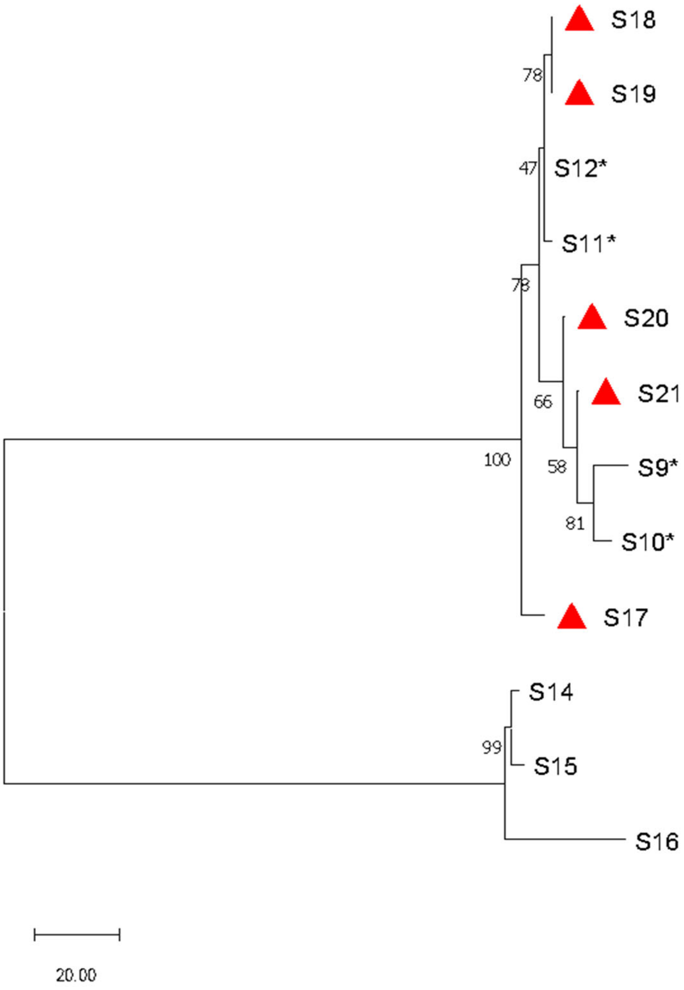

Figure S6. Unrooted phylogenetic tree inferred using the maximum parsimony analysis of partial *secY* gene sequences representative for each genotype (Tables S3. and S4.). The percentage of replicate trees in which the associated taxa clustered together in the bootstrap test (500 replicates) are shown above the branches. Scale bar representing phylogenetic distance is given in the units of number of

changes over the whole sequence. Genotypes present in Croatia are marked with an asterisk* and new genotypes, S17 – S21, are marked with red triangles.

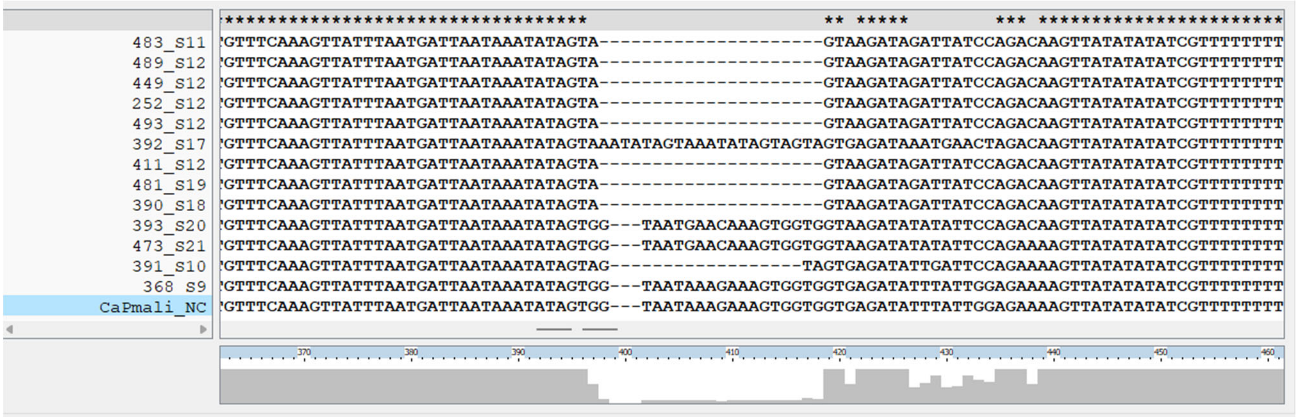

Figure S7. Alignment of representative nucleotide sequences for each *secY* genotype (Tables S3. and S4.). The figure shows a highly variable region (397-438/1248) within which, in comparison to the reference sequence of the *secY* gene from the AT strain of '*Ca*. P. mali', either an insertion (genotype S17, sample 392) or a deletion (genotype S12, e.g., sample 489) occurred.

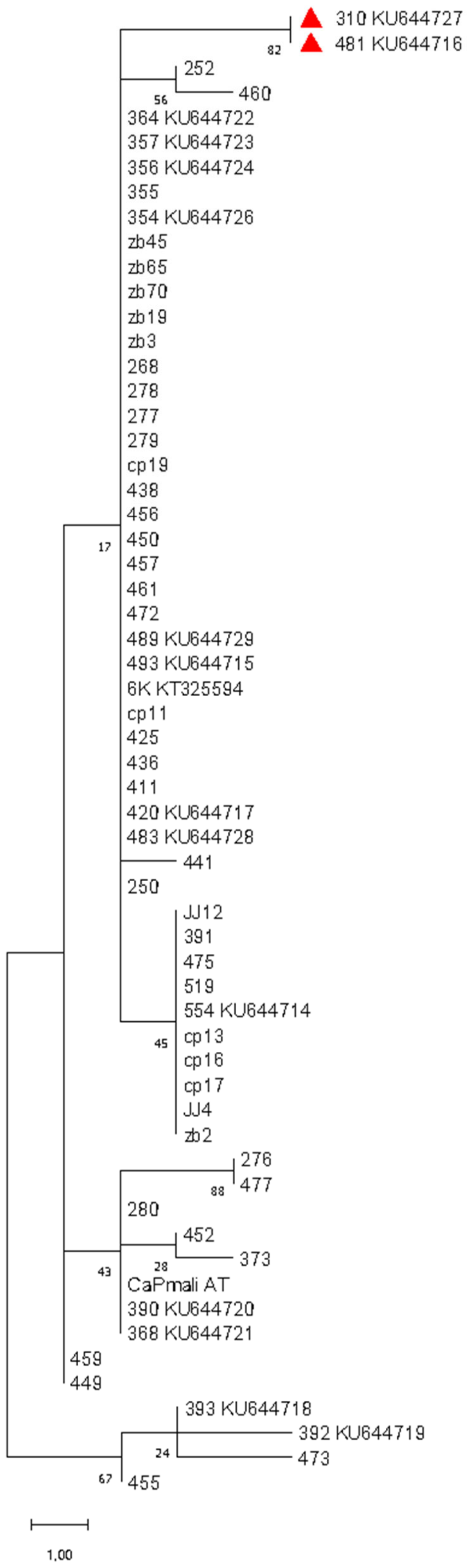

Figure S8. Unrooted phylogenetic tree inferred using the maximum parsimony analysis of complete *aceF* gene sequences (Tables S2. and S3.). The percentage of replicate trees in which the associated taxa clustered together in the bootstrap test (500 replicates) are shown above the branches. Scale bar representing phylogenetic distance is given in the units of number of changes over the whole sequence. Samples belonging to a novel A27 genotype are marked with red triangles.

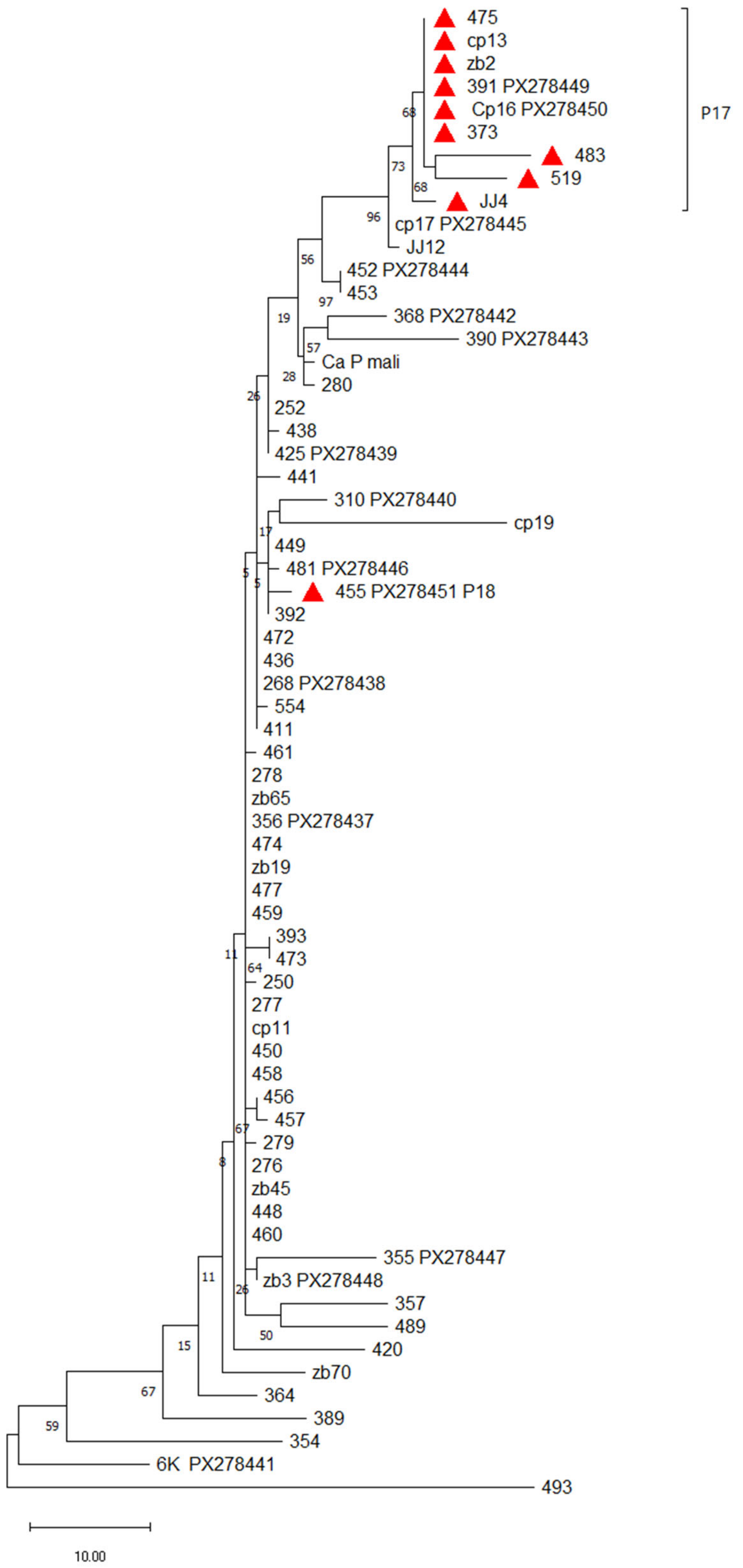

Figure S9. Unrooted phylogenetic tree inferred using the maximum parsimony analysis of complete *pnp* gene sequences (Table S2. and S3.). The percentage of replicate trees in which the associated taxa clustered together in the bootstrap test (500 replicates) are shown above the branches. Scale bar representing phylogenetic distance is given in the units of number of changes over the whole sequence. Samples belonging to novel P17 and P18 genotypes are marked with red triangles.

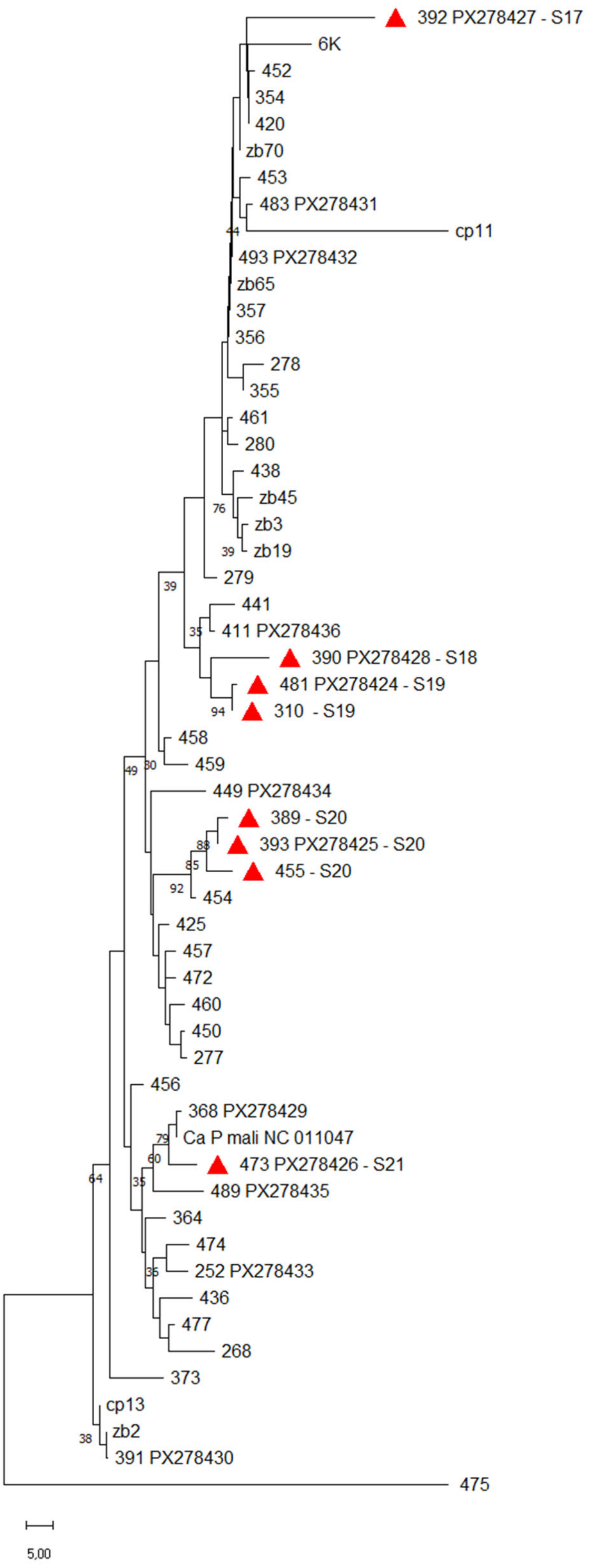

Figure S10. Unrooted phylogenetic tree inferred using the maximum parsimony analysis of complete *secY* gene sequences (Tables S2. and S3.). The percentage of replicate trees in which the associated taxa clustered together in the bootstrap test (500 replicates) are shown above the branches. Scale bar representing phylogenetic distance is given in the units of number of changes over the whole sequence. Samples belonging to novel S17 – S21 genotypes are marked with red triangles.
